# Peer support in type 2 diabetes: a randomised controlled trial in primary care with parallel economic and qualitative analyses: pilot study and protocol

**DOI:** 10.1186/1471-2296-8-45

**Published:** 2007-07-31

**Authors:** Gillian M Paul, Susan M Smith, David L Whitford, Eamon O'Shea, Fergus O'Kelly, Tom O'Dowd

**Affiliations:** 1 Department of Department of Public Health and Primary Care, Trinity College, Dublin 2, Ireland; 2 Department of General Practice, RCSI, Dublin 2, Ireland; 3 Irish Centre for Social Gerontology, National University of Ireland, Galway, Ireland; 4 Trinity College-Eastern Regional General Practice Training Programme, Health Services Executive, Dublin 2, Ireland

## Abstract

**Background:**

Diabetes is a chronic illness, which requires the individual to assume responsibility for their own care with the aim of maintaining glucose and blood pressure levels as close to normal as possible. Traditionally self-management training for diabetes has been delivered in a didactic manner. In recent times alternatives to the traditional delivery of diabetes care have been investigated, for example, the concept of peer support which emphasises patient rather than professional domination. This paper describes the pilot study and protocol for a study that aims to evaluate the effectiveness of a peer support intervention for people with type 2 diabetes in a primary care setting.

**Methods/Design:**

A pilot study was conducted to access the feasibility of a randomized controlled trial of a peer support intervention. We used the MRC Framework for the evaluation of complex interventions. Elements of the intervention were defined and the study protocol was finalized. In this cluster randomised controlled trial twenty general practices are assigned to control and intervention groups. Each practice compiles a diabetes register and randomly selects 21 patients. All practices implement a standardised diabetes care system. In the intervention group all practices recruit three peer supporters. The peer supporters are trained to conduct nine group meetings in their general practice over a period of two years. Each meeting has a structured component. The primary outcomes are blood pressure, total cholesterol, HBA1c and the Diabetes Well-being score. In addition to biophysical, psychosocial, economic and health service utilization data peer supporter activity and qualitative data are collected.

**Discussion:**

Peer support is a complex intervention and evaluating such an intervention presents challenges to researchers. This study will evaluate whether a peer support programme for patients with type 2 diabetes improves biophysical and psychosocial outcomes and whether it is an acceptable, cost effective intervention in the primary care setting.

**Trial registration:**

Current Controlled Trials ISRCTN42541690

## Background

### Improving outcomes in type 2 diabetes

The management of type 2 diabetes has traditionally focused on the lifestyle changes and medication that can improve blood sugar or glycaemic control. However, the United Kingdom Prospective Diabetes Study (UKPDS) and the Steno-2 study highlighted the importance of combining tight glycaemic control and cardiovascular risk reduction such as blood pressure control if long term complications are to be prevented or reduced [1,2]. Two Cochrane reviews of diabetes care concluded that care should be structured with regular prompting for patients and their family doctors; that multifaceted interventions can enhance professional performance and that the addition of patient-oriented interventions can improve outcomes [3,4]. Other research has focused on interventions to change diabetes-related behaviours or interventions that can enhance patient self-management [5,6].

However, more recent international studies failed to make significant impact on biophysical risk factors such as blood pressure, cholesterol and glycosylated haemoglobin (HBA1c) [7-10]. There is evidence that standards of diabetes care are suboptimal in many countries [11,12]. Questions remain as to the type or types of interventions that can significantly improve outcomes in individuals with type 2 diabetes.

### Peer support

Peer support has been formally defined as the "the provision of emotional, appraisal, and informational assistance by a created social network member who possesses experiential knowledge of a specific behaviour or stressor and similar characteristics as the target population, to address a health-related issue of a potentially or actually stressed focal person" [13]. This definition of peer support falls within the social support model, which is defined as the process through which social relationships might promote health and well-being [14]. A peer may have a greater understanding of an individuals situation than a member of their own family or social network who may feel uncomfortable about an issue or may be too upset to provide support [15]. Within the social support model, the direct effect model would postulate that peer support could reduce feelings of isolation and loneliness, provide information about access to health services or the benefits of behaviours that positively improve health and well-being and encourage more positive health practices [13].

Qualitative studies with people with type 2 diabetes have highlighted the value they place in learning from the experience of others [16] and the potential gap between knowledge and understanding, with a need for more "understandable information" in "layman's language" [17]. This could be addressed through a peer support intervention. A peer supporter is a type of lay health worker, who are defined in a Cochrane review as individuals who carry out functions related to health care delivery; are trained in some way in the context of an intervention and have no formal related professional or paraprofessional qualification [18]. The use of non-professional volunteers has improved outcomes in other diverse health settings such as maternal child health development [19] and cardiac surgery [20]. A participatory intervention using lay facilitators with womens' groups led to a significant reduction in neonatal mortality [21]. The dramatic success of this intervention has been attributed in part to the 'remarkable capacity and altruism' of the lay women facilitators but also to the fact that it was a well-targeted demand-side intervention [22].

Unlike the peer led educational interventions such as the Chronic Disease Self-Management Programme (CDSMP) [23] devised by Kate Lorig the intervention in this study focuses more on social support than education.

### Evaluation of peer support

Peer support is a complex intervention and ideally should be evaluated using a framework such as the Medical Research Council Framework for Development and Evaluation of Randomised Controlled Trials for Complex Interventions to Improve Health [24]. This framework highlights the importance of adequate modelling and exploration or piloting of the intervention prior to commencement of the full randomised controlled trial. It stresses the importance of comparing a fully defined intervention with an appropriate alternative and ensuring that the intervention can be replicated to facilitate long-term implementation. The importance of a process evaluation of a randomised controlled trial, which incorporates a qualitative component and includes a consideration of treatment fidelity, has also been emphasized [25-28]. We have used the MRC framework to develop the peer support intervention for this study and this process is described in detail elsewhere (submitted to BMC Health Services Research, in press). There is also increasing recognition of the importance of a parallel health economic analysis when carrying out a randomised controlled trial of an intervention with potential to improve health outcomes [29]. Costs must be considered when evaluating new treatments and this is particularly the case for a condition such as type 2 diabetes, which has enormous costs for individuals and healthcare systems [30].

## Methods/Design

### Pilot study

The pilot study was carried out in two general practices, one a small single-handed practice and the other a large group practice. Both practices have a practice nurse and computerized record systems. Twenty four patients and four peer supporters from the practices participated. Some characteristics of the participants are presented in Table 1. The peer supporters, who were selected by the GPs, attended two evening training sessions conducted by the research team. Each peer supporter facilitated three sessions with participating patients. These sessions were held in the general practices for approximately an hour and a half.

**Table 1:** Personal characteristic of the patients and peer supporters that participated in the pilot study

	**Patient participants**	**Peer supporters**
Male	13 (59%)	4 (100%)
Mean age (yrs)	66	65
Mean (yrs) since diagnosis of type 2 diabetes	4	7
Entitled to medical card	14 (64%)	2 (50%)
Smoker	3 (14%)	0 (0%)

The qualitative evaluation of the pilot study indicated that patients were positive about their experience of peer support-"very helpful because you are going into a hospital, seeing a doctor, but you are not seeing other people who have it like ourselves" (FG1, Participant 3). The patients were also positive regarding the professional diabetes education services but many felt that healthcare professionals may not be familiar with the day to day issues which arise when living with diabetes. Patients felt that a peer would serve as a motivating factor in both encouraging diabetes behaviours and in lifestyle modification. Peer supporters described the experience as "very positive" (FG7 Participants 4,5). They reported that the patients in their groups were interested in simple practical information- "the people are not looking for a theoretical understanding of it, you know they don't want to know the latin" (FG7, Participant 6). Potential barriers, such as confidentiality and intrusiveness were dismissed by patients and peer supporters. Payment was largely viewed as against the spirit of peer support though participants felt expenses should be covered. Practice staff reported that the patients appeared to enjoy the contact with each other and the study had little negative impact on their work or the day to day running of the practice.

These pilot results are in keeping with a recent UK study looking at the role of lay health advisers in diabetes [31]. Health professionals and patients were interviewed and both were broadly supportive of the concept of lay advisors following the development of lay criteria, structured training and guidelines. Patients indicated a preference for a lay advisor over an unknown health professional and they identified the ability of lay advisors to enhance and support diabetes patient self-management. Several peer support or 'buddy' projects are being piloted in the UK though none are currently being evaluated using a rigorous methodology such as a randomised controlled trial [32].

### Randomised controlled trial

#### Objective

To determine whether a peer support programme for patients with type 2 diabetes improves biophysical and psychosocial outcomes and whether it is an acceptable, cost effective intervention in the primary care setting

The study is a cluster randomised controlled trial where general practices are allocated into one of two groups:

1. Control practices: implement a standardised diabetes care system for the 21 study patients (see below).

2. Intervention practices: implement the standardised diabetes care system and the peer support intervention (see below)

#### Identification and recruitment of practices and patients

##### Practice identification

Twenty practices will be identified from the Trinity College/Health Service Executive General Practice Training Programme and the Trinity College network of undergraduate teaching practices. They all have a practice nurse and vary from single-handed practices to multi-partner practices with the largest practice having five full time equivalent partners. They include a mixture of urban, suburban and rural practices.

The GP's are provided with information about the study. They are then contacted by the research team and asked if they are interested in participating in the study. If they agree to participate the project manager visits the practice and discusses the study in more detail. During this meeting the project manager gets commitment to the study and one GP and practice nurse are identified as managing the programme at practice level. Participating practices are given a grant of €5000 per annum for three years (€1000 of which is allocated to the practice nurse). Practices are eligible to participate if they have a practice nurse; have computerized records; did not participate in the pilot study; have a minimum GMS (General Medical Services scheme, see Table 2) list of approx 1000 patients and/or have > 50 patients on their register of patients with type 2 diabetes; and are not participating in an existing shared care diabetes programme involving structured care between the general practices and hospital diabetes service.

**Table 2:** The General Medical Services scheme

THE GENERAL MEDICAL SERVICES SCHEME [33]
-Free medical and surgical health services for those on low income and for everyone over 70 yrs of age
-An eligible person is entitled to select a General Practitioner of their choice, from those doctors who have entered into agreements with the Health Service Executive
-29.5% of the population are entitled to a medical card

##### Patient identification (21 patients per practice)

Participating practices compile a list of eligible patients from their practice to create or update their diabetes register. A combination of approaches to compile the register are used including personal recollection of practitioners, identification of patients through computerized prescribing of diabetes related medications and if necessary using computerized records of local pharmacies. Diagnosis of type 2 diabetes is confirmed by double-checking patient records. Only patients agreeing to participate in the intervention and who provide baseline data collection will be recruited into the trial. Details of those declining to participate will be recorded including age, gender, GMS status, duration of diabetes and diabetes treatment. A patient is eligible to participate if they are over 18 years of age; have type 2 diabetes and attend participating practices. A patient will be excluded if they have significant mental or physical illness which is likely to impair their capacity to participate in the programme

#### Randomisation

##### Practice allocation

Practices will be stratified by practice size (less than two or two or more whole-time-equivalent GPs) and presence of existing structure primary diabetes care service. They will then be allocated to intervention or control groups using minimization [34]. Allocation will be carried out independently of the research team. It will occur prior to patient identification and baseline data collection. While it is regarded as best practice to randomize after baseline data collection, in this study allocation must take place in this sequence to facilitate the recruitment of peer supporters from the patient register in intervention general practices before the randomisation of patients and the beginning of the intervention.

##### Patients: random selection

**Figure 1:**
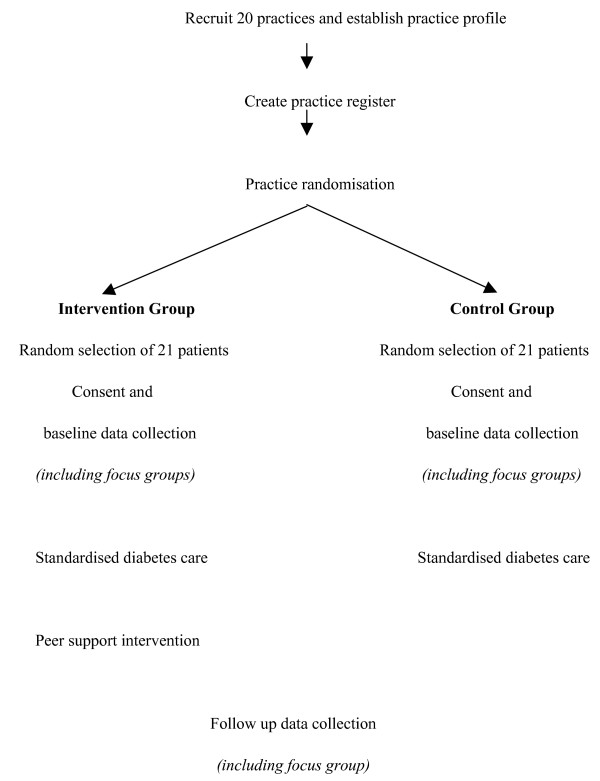
Flow of practices and patients throughout the trial.

Once a complete diabetes register is established, the project manager assists the practices in using a list of random numbers to select patients for participation. All patients on the register are allocated a number 1 to Y (where Y is the total number of patients on the list). Randomly selected patients are sent an invitation letter, information sheet and reply slip. Patients who do not respond in two weeks are contacted by telephone by the practice nurse to ascertain interest in participation. If a patient does not meet the eligibility criteria or declines to participate the next patient is selected from the list until the quota of 21 is reached. (Figure 1)

Basic characteristics of patients who decline to participate will be collected including date of birth, sex, GMS status, duration of diabetes and reason for not participating.

#### Sample size calculation

The study aims to achieve a sample of 410 patients from 20 practices. This takes into account the effect of cluster randomisation and allows for 80–85% patient follow up and a 15% practice attrition rate (though the authors previous experience of cluster randomized trials in this setting indicate much lower patient and practice attrition rates [10]). Sample size calculations are based on a 20% improvement from baseline in the control group and 50% improvement in the intervention group [35]. All calculations are two-sided and based on an alpha of 5% and a power of 80%.

##### Systolic Blood pressure (SBP)

46% of patients, in a previous study in Dublin, have a SBP >160 mmHg [10]. This level of SBP represents very poorly controlled blood pressure as the current upper recommended limit for an individual with type 2 diabetes is 130 mmHg [36]. The intracluster coefficient (ICC) for SBP is 0.001 [10]. A sample size of 400 patients from 20 practices is needed to show a significant improvement in the proportion of patients with a SBP below 160 mmHg (i.e. 50% improvement in intervention group and 20% improvement in control group as above).

##### HBA1c

A sample size of 130 patients from 8 practices is needed to demonstrates a clinically significant difference in mean HBA1c between intervention and control groups (i.e a difference of 0.9% [37], standard deviation 1.6; ICC 0.001 [10])

##### Cholesterol

57% of patients have a total cholesterol >5 mmol [10], which is the upper recommended limit for patients with type 2 diabetes [38] (ICC = 0.06) [10]. A sample size of 410 patients from 20 practices is needed to show a significant improvement in the proportion of patients with a cholesterol <5 mol/l.

##### Well-being scores (WBS)

A sample size of 221 patients from 12 practices is needed to show a clinically significant difference in WBS between intervention and control groups (i.e. a mean difference of 5 points, standard deviation, 10.3; ICC of 0.07) [10].

#### GP and Practice nurse training

The GP's and practice nurses receive a practice based training session. This 1.5 hour session is conducted by a GP on the research team. The content focuses on the treatment of type 2 diabetes in primary care.

Practice nurses attend three training sessions conducted by the research team. The sessions are outlined below:

##### Session 1 (Prior to baseline data collection)

Introduction to project

Random selection of patients from register

Recruitment of participants: letter, phonecall, details of non-responders

Data collection: Information to give to potential participants

Informed consent

Questionnaire completion

Performing biophysical measurements

Confidentiality

Computer skills/imputing data

##### Session 2 (Prior to the commencement of the intervention)

Diabetes care in primary care (including annual audit)

Setting up a recall system

##### Session 3 (Prior to follow up data collection)

Refresher of data collection procedures

#### Standardised diabetes care

All participating practices are trained and supported to introduce a standardised primary diabetes care system. This is to avoid the lack of clarity that can result if 'usual care' is used for the control arm of a randomised controlled trial. This diabetes care system includes:

• Practice based training for GPs and practice nurses (as above)

• Agreement and implementation of evidence based clinical guidelines

• Structured registration and recall every four months of patients with type 2 diabetes to specific diabetes appointments or mini-clinics with practice nurses supported by GPs

• Provision of treatment algorithms designed to optimise glycaemic control and reduce cardiovascular risk

• Use of a 'target card', a patient held record of results relating to their diabetes

• Educational resources

• Annual practice audit

Practices with existing structured care (three of the twenty), have agreed to incorporate these components into their current system

#### Intervention

Intervention practices, deliver a peer support intervention which has the following components:

##### 1. Peer supporters

Potential peer supporters are identified by GPs and practice nurses in the intervention practices. Peer supporters are recruited and trained at a ratio of approximately one peer supporter to seven/eight patients with type 2 diabetes. They are eligible to be trained if they meet the following inclusion criteria:

• They have had type 2 diabetes for at least one years duration

• They participate in preventive treatments and are generally adherent to treatment and behaviour change regimens, as judged by the practice team

• They have a capacity and commitment to undergo the training required (outlined below)

• They have a full understanding of the importance of patient confidentiality

• They undertake to liaise with the practice nurse and/or GP if unanticipated problems arise during the course of their peer support activity

##### 2. Peer supporter training

The peer supporters attend two evening training sessions, which are conducted by the research team. The content of these sessions is outlined below:

###### Session 1

Introduction to the project

Role of the peer supporter

Basics of type 2 diabetes

Complications of type 2 diabetes

###### Session 2

Lifestyle and medication issues

Communication skills and working with groups

Role play

Confidentiality

Support for the peer supporters

The two sessions will focus on the materials to be used during the group meetings (described below) and peer supporters will each receive a pack with resource material to support these training sessions.

##### 3. Retention and support of peer supporters

Retention of peer supporters is crucial to the study. Structures are in place to ensure peer support workers are supported in the role. They include:

• Feasible time commitment to the project

• Outline of responsibilities/peer support policy

• Adequate training (outlined above)

• Course handbook and resource pack

• Contact details and explicit support from the project team and GP/practice nurse

• Telephone call from project manager following each session

• Annual social event

• Protocol to follow if a peer supporter resigns (see below)

• Travel and related expenses

If a peer supporter resigns from their position a substitute will be nominated from their group. At the beginning of the intervention the peer supporters and their groups will be asked to nominate one group member who would be a suitable replacement for him/herself if the need were to arise. An individual will receive the same training as the original peer supporters if they need to take on the full role of peer supporter. The peer support intervention operates within a volunteer framework, which involves reimbursement for travel and communication expenses rather than actual payment for work involved.

##### 4. Peer support meetings

Peer support meetings are held in the general practice premises at a convenient time for practice staff, peer supporters and participants. The intervention consists of nine peer support sessions held over two years; at month 1, month 2 and every 3 months thereafter. The exploratory phase revealed that both peer supporters and patients agree that each meeting should have a focus and a small structured component, for example, a ten to fifteen minute discussion on a particular aspect of diabetes management (see Table 3 for a summary of the meeting content. Peer supporters receive more information including a list of potential questions to put to the group on each topic). A need was also identified for a "frequently asked questions" system. That is, at the end of each session there is general discussion and the group identifies and records any questions regarding the session topic. These are fed back to the research team who compile written answers based on the feedback from all groups, which are presented and discussed at the start of the next session.

**Table 3:** Content of peer support meetings

***MEETING 1***	***MEETING 2***
***INTRODUCTION***	***HEART AND VASCULAR DISEASE***
• Introduction to each other • What is peer support? • Ground rules • Discussion on course content (9 sessions) • Video/DVD 15 mins • Entitlements in diabetes • Identifying a substitute peer supporter • Contact details for the group	• Why is it so important? • How you can reduce your risk of heart disease and other vascular complication ○ Hypothetical individual and what they would advise them to do Questions relating to heart disease including blood pressure and cholesterol medication and taking tablets
***MEETING 3***	***MEETING 4***
***BLOOD SUGAR LEVELS***	***HEALTHY EATING***
• Information on hypo/hyperglycaemia • Blood sugar testing Questions on blood sugar levels What to do when you are sick	Discussion of healthy 'eating plate' • Laminated picture of the 'healthy plate' Healthy eating quiz and discussion of answers Questions on healthy eating in diabetes

***MEETING 5***	***MEETING 6***
***MEDICATION***	***EXERCISE***
• Control of type 2 diabetes ○ Diet ○ Tablets ○ Insulin Questions regarding medication including side effects	• Importance of exercise • Use of a pedometer ○ each person will be given a pedometer Questions about exercise Maybe arrange a walk in locality

***MEETING 7***	***MEETING 8***
***FOOT CARE***	***EYE AND KIDNEY COMPLICATIONS***
• Why foot care matters in diabetes • Discussion on how to check feet ○ Laminated sheet to cover all aspects of foot care Questions relating to the feet Information on local chiropody services	• What happens to the eyes and kidneys in diabetes • Importance of good blood pressure and blood sugar control in order to prevent complications Questions relating to eye and kidney disease

***MEETING 9***	
***LIVING WITH DIABETES***	
This is intended to be a relatively open session in which the group can discuss any remaining concerns and consider whether they would like to continue to meet Importance of follow up data collection!	

#### Informed consent and ethical approval

An invitation and information sheet is sent to the randomly selected eligible patients. Patients are asked to sign and return a reply slip if they are interested in participating. They are contacted by the practice nurse and an appointment is made for them to visit the practice for baseline data collection. Prior to baseline data collection the practice nurse reads the information sheet to the patient, allowing them to ask any questions and confirming their willingness to participate in the study. The practice nurse then asks the patient to sign the consent form.

Ethical approval has been obtained from the Ethics Committee of the Irish College of General Practitioners (Protocol No.: REC0904-11; 01/12/04)

#### Data collection

Data collection is carried out in the practices by practice nurses, following training by the project manager. Data are collected at baseline and on study completion at two years. The following forms of data are collected:

1. Questionnaire: given to patients as they wait to see the practice nurse. It is then checked for completeness of data entry and the nurse assists any patient who requires help in completing the questionnaire measures

2. Consultation and biophysical data: gathered by practice nurse

3. GP record search: to establish process of care and collect patient outcomes recorded in GP records, carried out by practice nurse.

4. Peer support activity: Peer supporters will keep a diary of their activity (outlined below)

##### Questionnaire

The questionnaire includes:

• *Personal and demographic variables*

○ Name, date of birth, marital status,

○ Educational level

• *Economic variables*

○ Occupation

○ General Medical Services eligibility

○ Journey times to GP practice and hospital

• *Health service utilization*

○ Number of visits to GP practice (recorded by practice nurse)

○ Number of visits to diabetes day centre (recorded by patient)

○ Number of visits to OPD to see a specialist (recorded by patient)

• *Peer support utilisation*

○ Peer support contacts: number, nature, duration (recorded by peer supporter)

• *Psychosocial*

○ Well-being score [39]

○ Diabetes self-care activities [40]

○ Self-efficacy [41]

○ Measure of medication adherence [42]

○ Family and friends subscale of the Chronic Illness Resources Survey [43]

##### Consultation and biophysical data

The following biophysical measurements are collected:

• Blood Pressure

• HBA1c

• Cholesterol: Total random cholesterol

• Body mass index

##### GP record search

Patient charts are searched by the practice nurse and process of care information recorded:

• Number of GP visits

• Number of practice nurse visits

• Patients medical history: disease counts to establish multimorbidity

• Patients medication

##### Peer support activity

Peer supporters fill out a diary of their peer support activity after each peer support meeting. They document the following information:

• Number of peer support meetings

• Nature of meeting

○ Number and names of people attending

○ Topics discussed

○ Problems arising

• Time commitment to study

### Data analysis

All results will be analysed using STATA statistical software [44] which has a facility for complex survey sample analysis that allows for adjustments in data analysis based on design effects, planned or unplanned. Analyses will be carried out using two approaches:

• by intention to treat i.e. including all randomized patients, regardless of their participation in the intervention.

• Sensitivity analyses will explore whether adherence to the intervention influences the effect of the intervention on primary outcomes

Participating patients will be described in terms of:

• Age

• Gender

• Date of diabetes diagnosis

• Single/Multimorbidity

• Socioeconomic status

Practices will be described as follows:

• Number of GPs (WTE)

• Number of practice nurses (WTE)

• Number of administrative staff (WTE)

• Practice size (No of GMS patients and total number of patients)

• Practice geographical location

#### Randomised control trial analysis

##### Primary outcomes

• Blood pressure

• Total cholesterol

• HBA1c

• Well being score

##### Secondary outcomes

• Biophysical

○ BMI

• Measure of processes of care

○ GP visits

○ Practice nurse visits

○ Hospital OPD visits

○ Hospital diabetes centre visits

○ Hospital admissions

• Psychosocial measures and medication adherence

○ Diabetes self-care activities

○ Self-efficacy

○ Measure of medication adherence

○ Smoking

• Medication

○ Aspirin

### Data management

The project manager has the overall responsibility for compilation, maintenance and management of the study database. The database is stored on a password-protected computer in a locked office.

Practice nurses receive training in data collection and have responsibility for completeness, accuracy and confidentiality of the data within participating practices. Data are entered onto a study laptop, onto a File Maker Pro [45] file by the practice nurse in participating practices. Study files are password protected. Each patient has a unique study number. The data are collected by the project manager and merged with the main study database. The data from each practice are merged, checked, cleaned and transferred into STATA.

The project manager double checks the questionnaires and patient records in each practice to ensure data collection and entry is accurate and complete.

### Treatment fidelity

Treatment fidelity is the strategy used to monitor and enhance the reliability and validity of behavioural interventions [46]. This study uses elements of a framework developed by Bellg et al to make treatment fidelity explicit [27]. The framework consists of five treatment fidelity strategies: Treatment design, Training procedures, Delivery of treatment, Receipt of treatment and Enactment of treatment skills. Details of how the strategy is implemented in this study are presented in the Table 4.

**Table 4:** Framework of treatment fidelity strategies

Treatment design	Information provided about intervention *Length of peer support meetings (1 to 1.5 hrs)* *Number of peer support meetings (9)* *Duration of intervention (2 yrs)* Information provided on standardized care *Number of routine visits to GP/practice nurse (every 4 months)*
Training procedures	Training of GP and practice nurse outlined *Practice based session for GPs and practice nurses* *Three training sessions for practice nurses* Training of peer supporters outlined *Two evening sessions*
Delivery of treatment	Assurance that intervention has been delivered as per protocol *Peer supporter manual* *Project manager contacts every peer supporter after each of their group meetings* *Peer supporters fill in log diaries* *Focus groups with participants, peer supporters and professionals*
Receipt of treatment	Record of each participant's attendance at meetings *Peer supporter takes a record at each meeting*

### Qualitative evaluation

A descriptive parallel qualitative analysis is being carried out to record patients, professionals and peer supporters attitudes to peer support and their experience of its delivery in intervention practices. It will be based on descriptive phenomenology. Descriptive phenomenology is one of several qualitative research traditions. It seeks to understand the lived experience of individuals. It answers questions such as "What is it like to have a certain experience?"[47]. It is becoming increasingly relevant to health service research as it provides insight into service users and providers' experiences and stories [48].

In the intervention group the qualitative evaluation will explore questions such as

1. What are the current support and resources for people with type 2 diabetes?

2. Peer support

• What is the participants' understanding of peer support?

• Is peer support an acceptable intervention?

• How does peer support work?

• Was the intervention successfully implemented?

• Did the programme benefit the peer supporters?

• Will people take up this service in the future if it was offered?

3. Standardised diabetes care programme

• Was the standardised diabetes care programme successful?

• Were patients satisfied with the programme?

4. Did the intervention and standardised diabetes care programme significantly increase the practice staff's workload?

In the control group the qualitative evaluation will explore questions such as:

1. What are the current support and resources for people with type 2 diabetes?

2. Standardised diabetes care programme

• Was the standardised diabetes care programme successful?

• Were patients satisfied with the programme?

3. Did the standardised diabetes care programme significantly increase the practice staff's workload?

#### Qualitative data collection and analysis

Data will be collected at baseline and follow up. Both focus groups and semi-structured interviews will be conducted among patients, peer supporters and practice staff:

• Patients (4 focus groups)

• Peer supporters (2 focus groups)

• GPs (4 semi structured interviews)

• Practice nurses (4 semi structured interviews)

Stratified purposeful sampling will be used in order to include patients, peer supporters and staff from both urban/rural practices, small/large practices and from deprived and affluent areas. Patients and peer supporters will be invited to participate in the focus groups by letter, which will be followed up by a telephone call from the practice nurse. Staff will be invited to participate by the project manager.

##### Interview schedule

The interviews and focus groups will be audio-taped and transcribed verbatim.

The analysis will involve reading the transcripts, identification and transforming meaning units, formulation of essential general structure or structures and finally formulating the exhaustive description of the phenomenon. Several methods of improving validity of qualitative research will be conducted including respondent validation, clear explanation of methods of data collection and analysis, reflexivity and audit trails. The software package, Atlas, will be used to assist with organization and analysis of the data.

#### Process evaluation

Data from the peer supporter log diaries and the project manager's record of contact with the peer supporters is analysed, in addition to the above qualitative data, to evaluate the process of the implementation of the intervention.

#### Economic evaluation

The economic evaluation will examine whether the intervention is associated with overall cost increases or cost decreases and will link the cost changes to incremental gains in effectiveness and primary outcomes. The basic tasks of the economic evaluation are to identify, measure, value and compare the costs and outcomes of the addition of peer support to a standardised primary diabetes care system. Unit costs will be applied to the resource use data to calculate the various costs of care. The primary focus will be on cost effectiveness analysis [49]. Presenting the results of cost effectiveness studies requires reporting the incremental costs and effects of a new intervention relative to an alternative and then reporting an incremental cost effectiveness ratio (incremental costs divided by incremental effects). In this study the cost effectiveness analysis will provide information on the marginal costs and effects of the intervention relative to the alternative through the calculation of incremental cost-effectiveness ratios using the primary outcomes: blood pressure, total cholesterol, HBA1c and diabetes well-being. Incremental cost-effectiveness ratios will be calculated in all circumstances, except where equivalence in effect has been proven between intervention and control group [50].

Costs are likely to fall on patients, their families and the state. The health care resources consumed will reflect the costs of organising and operating peer support in addition to standardised primary diabetes care. These costs will reflect the time input of health professionals and fixed or overhead costs, including any new equipment and capital expenditure. All contacts of patients with the health services will be recorded and valued, including general practice use, hospital attendances (both outpatient clinic and diabetes centre visits), admissions and drug prescriptions. Travel and time costs will also be calculated. The cost of the peer support system will be a major component of overall intervention costs. Estimates will be made of the work and leisure time foregone by peer support workers and monetary valuations will be estimated as part of the opportunity cost methodology. Training costs will also be measured and valued. Patient and family costs will include any out-of-pocket expenses and any own time input into the treatment process, evaluated using an opportunity cost methodology.

## Discussion

Diabetes self management training has traditionally been delivered in a didactic manner with emphasis on imparting knowledge. However this approach has been shown to be ineffective in changing the behaviour of individuals with type 2 diabetes and improving metabolic control [51]. An alternative approach such as peer support may improve these outcomes. This approach is based on a social support theoretical framework rather than an educational or psychological framework and the outcomes we have selected for the study reflect this underlying theoretical framework. The potential advantage of peer support is that it focuses on the impact of the illness on daily life rather than on medical information about illness. The group meeting components of the intervention in this study are designed to build on the lay element of peer support through the encouragement of the sharing of experiences and exchanges between participants. Peer support is a complex intervention and a strength of this study has been the use of the MRC Framework for the evaluation of randomized controlled trial of complex interventions designed to improve health outcomes [24]. Details of the initial phases of the framework leading to the finalisation of this protocol are presented elsewhere. (BMC Health Services Research, in press). The study is specifically based in the general practice setting and empowers and supports practice staff in the recruitment of participants and peer supporters and data collection. This approach embeds the study in the real world of primary care and will increase the generalisability of results. An additional strength of the study is its duration of two years, which allows us to test the sustainability of a peer support intervention in a clinical setting over time.

Peer supporters are central to the study and methods of supporting them in their role have been identified. The project manager is a source of advice and support for peer supporters. They are also encouraged to turn to their GP and practice nurse who are the clinicians responsible for the care of all patients within each peer support group.

In summary, peer support is a complex intervention and evaluating such an intervention presents challenges to researchers. This study aims to determine whether a peer support programme for patients with type 2 diabetes improves biophysical and psychosocial outcomes and whether it is an acceptable, cost effective intervention in the primary care setting.

## Competing interests

The author(s) declare that they have no competing interests.

## Authors' contributions

All authors reviewed and approved the final version of this manuscript. GP, SMS, TOD, EOS and DW designed the study, prepared the protocol and participated in writing this paper. FOK prepared the protocol and participated in writing the paper.

## Pre-publication history

The pre-publication history for this paper can be accessed here:


